# A Case Report of Rheumatoid Arthritis With a Migratory Pattern

**DOI:** 10.7759/cureus.86677

**Published:** 2025-06-24

**Authors:** Nima Sadeghi, Brayden Haberman, Jamie McDermott, Nathan Matthews

**Affiliations:** 1 Medicine, Midwestern University Arizona College of Osteopathic Medicine, Glendale, USA; 2 Medicine, Creighton University School of Medicine, Phoenix, USA; 3 Family Medicine, HonorHealth, Mesa, USA

**Keywords:** anti-ccp antibodies, case report rheumatology, migratory arthralgia, migratory polyarthritis involving large joints (60-80%), rheumatoid arthritis

## Abstract

Rheumatoid arthritis (RA) is a systemic autoimmune disease that classically presents with symmetric polyarthritis. However, atypical manifestations such as migratory joint pain can obscure diagnosis, particularly in patients with pre-existing degenerative joint conditions. We report the case of a 74-year-old woman with a history of osteoarthritis (OA), obesity, and multiple comorbidities who developed acute, migratory joint and muscle pain over several weeks. Her symptoms exhibited a shifting pattern, initially involving the left wrist and groin, before progressing to the right upper extremity with notable swelling and functional impairment. Laboratory evaluation revealed a positive anti-cyclic citrullinated peptide (anti-CCP) antibody and elevated inflammatory markers, including C-reactive protein (CRP) and erythrocyte sedimentation rate (ESR). Rheumatoid factor (RF), antinuclear antibodies (ANA), and antineutrophil cytoplasmic antibodies (cytoplasmic (c-ANCA), perinuclear (p-ANCA), and atypical p-ANCA) were negative. Imaging studies excluded acute structural or vascular pathology. Despite her overlapping OA symptoms and seronegativity for RF, the patient’s clinical trajectory and positive anti-CCP supported a diagnosis of RA with an atypical, migratory presentation. She experienced partial symptomatic improvement with corticosteroids and was referred to rheumatology for initiation of disease-modifying antirheumatic drugs (DMARDs). This case underscores the need to consider RA in the differential diagnosis of migratory arthralgia, especially in older adults with existing joint disease, and highlights the diagnostic utility of anti-CCP antibodies in seronegative presentations.

## Introduction

Rheumatoid arthritis (RA) is a chronic autoimmune inflammatory disease that primarily affects the synovial joints, leading to progressive inflammation, joint deformity, and cartilage destruction [1]. RA typically presents as symmetric polyarthritis, but it can occasionally exhibit a migratory pattern that mimics other inflammatory arthritides, thereby delaying diagnosis [2]. Patients often report morning stiffness, polyarticular joint pain, and systemic symptoms such as fatigue and weight loss [1]. RA can also involve multiple organ systems, contributing to significant morbidity and mortality, primarily due to infections, cardiovascular disease, and pulmonary complications [1-3].

The diagnosis of RA is clinical, supported by a combination of laboratory markers and imaging studies [4]. Inflammatory markers such as C-reactive protein (CRP) and erythrocyte sedimentation rate (ESR) are often elevated, and serologic tests, including rheumatoid factor (RF) and anti-cyclic citrullinated peptide (anti-CCP) antibodies, help confirm the diagnosis [4-6]. However, a subset of patients may present with seronegative RA, making diagnosis more challenging [5]. Given the broad spectrum of presentations, the differential diagnosis includes inflammatory bowel disease (IBD)-associated arthritis, infectious arthritis, reactive arthritis, systemic lupus erythematosus (SLE), and osteoarthritis (OA) [4-5].

Although there is no cure for RA, disease-modifying antirheumatic drugs (DMARDs), particularly methotrexate, are the cornerstone of treatment, helping to slow disease progression and improve quality of life [5]. Adjunctive therapies such as non-steroidal anti-inflammatory drugs (NSAIDs) and corticosteroids provide symptomatic relief, while lifestyle modifications including smoking cessation, regular exercise, and dietary adjustments also play an essential role in disease management, as smoking is associated with worse clinical outcomes and increased disease severity [4,5].

The etiology of RA is multifactorial, involving a complex interplay of genetic predisposition, female sex, increasing age, and environmental triggers [3-5]. Although the pathogenesis is not fully understood, it involves dysregulated immune responses, autoantibody production, and chronic synovial inflammation [1,3]. Early recognition and appropriate treatment are critical to preventing irreversible joint damage and minimizing systemic complications [1-3].

## Case presentation

A 74-year-old woman with a past medical history of allergic rhinitis, hypertension (HTN), hypothyroidism, benign paroxysmal positional vertigo (BPPV), environmental allergies, type 2 diabetes mellitus (not requiring long-term insulin use), Class I obesity (BMI 33.28 kg/m²), and osteoarthritis (OA) presented to the outpatient clinic for evaluation of migratory joint and muscle pain. Her surgical history included cholecystectomy, endometrial ablation, thyroid surgery, and prior fracture repair. Home medications included albuterol as needed, azelastine nasal spray, cetirizine, diltiazem + spironolactone, and levothyroxine.

Approximately three weeks prior to presentation, the patient developed left wrist discomfort, followed two days later by left groin pain. One week after symptom onset, she developed acute right biceps pain, severe enough to prevent her from lifting her arm. She presented to the emergency department, where imaging of the right shoulder showed no acute fractures or dislocations. She was diagnosed with adhesive capsulitis and prescribed prednisone 20 mg and tramadol, neither of which alleviated her symptoms.

In the subsequent days, she developed acute right groin pain that resolved overnight, only to be replaced by worsening right upper arm pain. Her symptoms then continued to migrate, affecting the left thenar eminence, left hip, and again the right biceps. Approximately 10 days prior to her clinic visit, she noted new swelling and tenderness throughout the right upper extremity, extending from the shoulder to the hand. She experienced reduced range of motion and significant functional limitations. On the physical exam, shoulder abduction was limited to 70 degrees (normal: 150-180 degrees). Additional findings included left wrist tenderness with swelling, diffuse swelling of the right arm and right hand, and swelling in the left wrist and hand with mild tenderness to palpation. Diagnostic laboratory and imaging studies were ordered to identify the cause of her symptoms and included a complete blood count (CBC), rheumatologic and immunologic panels, and radiographic and CT imaging of the right upper extremity. She was prescribed tramadol hydrochloride (HCL) 50 mg QID PRN for management of pain until results from her initial testing could be reviewed and a more targeted treatment plan could be initiated.

Laboratory evaluation (Table 1) revealed a weakly positive anti-cyclic citrullinated peptide (anti-CCP) IgG antibody titer of 17 units (normal: ≤16 U/mL), a mildly elevated CRP of 0.9 mg/dL (normal: ≤0.5 mg/dL), and an ESR of 24 mm/hr (normal: 0-36 mm/hr). CBC was notable for a mildly elevated eosinophil count (0.6 K/μL) and monocyte count (0.87 K/μL; normal range: 0.26-0.80 K/μL); hemoglobin and white blood cell counts were within normal limits. Uric acid was normal. RF, ANA, and ANCA panels were all negative.

**Table 1:** Results of CBC and Autoimmune Panel WBC: white blood cell count; RBC: red blood cell count; MCV: mean corpuscular volume; MCH: mean corpuscular hemoglobin; MCHC: mean corpuscular hemoglobin concentration; RDW-SD: red cell distribution width standard deviation; RDW-CV: red cell distribution width coefficient of variation; MPV: mean platelet volume; nRBC: nucleated red blood cell (relative count); CRP: C-reactive protein; ANA Screen, IFA: anti-nuclear antibody screen by immunofluorescence assay; C-ANCA: cytoplasmic anti-neutrophil cytoplasmic antibody; P-ANCA: perinuclear anti-neutrophil cytoplasmic antibody; PR-3: anti-proteinase 3; CCP: cyclic citrullinated peptide; RF: rheumatoid factor.

Test	Patient Value	Reference Range	Units
WBC	9.6	4.0-10.9	10^3^/µL
RBC	4.79	3.50-5.40	10^6^/µL
Hemoglobin	14.2	12.0-16.0	g/dL
Hematocrit	43.9	36.0-48.0	%
MCV	91.6	80.0-98.0	fL
MCH	29.6	27.0-34.0	PG
MCHC	32.3	31.0-37.0	g/dL
RDW-SD	45.5	36.4-46.3	fL
RDW-CV	13.3	11.5-14.5	%
Platelets	254	130-450	10^3^/µL
MPV	9.7	7.4-12.4	fL
Lymphocytes	33	-	%
Lymphocytes absolute	3.21	0.90-3.50	10^3^/µL
Monocytes	9	-	%
Monocytes absolute	0.87	0.26-0.80	10^3^/µL
Eosinophils	7	-	%
Eosinophils absolute	0.66	≤0.62	10^3^/µL
Basophils	1	-	%
Basophils absolute	0.05	≤0.10	10^3^/µL
Neutrophils	50	-	%
Neutrophils absolute	4.82	1.48-8.32	10^3^/µL
Immature granulocytes	0	-	%
Immature granulocytes absolute	0.03	0.00-0.10	10^3^/µL
Nucleated RBC absolute	0	0.000-0.020	10^3^/µL
nRBC	<1.0	-	%
Sed Rate	24	0-36	mm/hr
CRP	0.9	≤0.5	mg/dL
Uric acid	5.8	2.6-6.0	mg/dL
ANA screen, IFA	Negative	Negative	-
C-ANCA	<1:20	Negative:<1:20	titer
P-ANCA	<1:20	Negative:<1:20	titer
Atypical P-ANCA	<1:20	Negative:<1:20	titer
PR-3 absolute	<0.2	0.0-0.9	units
CCP IgG absolute	17	≤16	U/mL
RF	<8.0	<8.0	IU/mL

**Figure 1:**
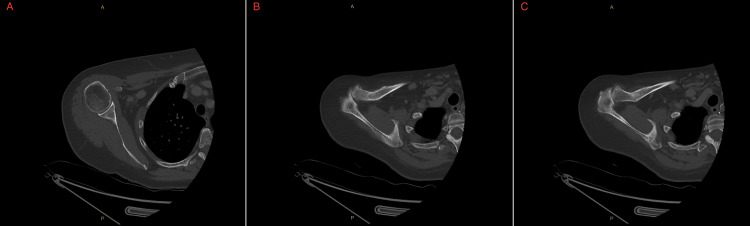
Axial CT Bone Window of the Humerus With IV Contrast Axial CT images of the right humerus in bone window (with IV contrast), demonstrating key osseous findings. (A) Axial view through the glenohumeral joint shows mild-to-moderate joint space narrowing and subchondral sclerosis, consistent with glenohumeral osteoarthritis. No fracture, dislocation, or effusion is seen. (B, C) Axial views through the acromioclavicular joint demonstrate moderate-to-severe joint space narrowing, osteophyte formation, and cortical irregularity, consistent with advanced acromioclavicular osteoarthritis. No osseous lesions or periarticular collections are present.

**Figure 2:**
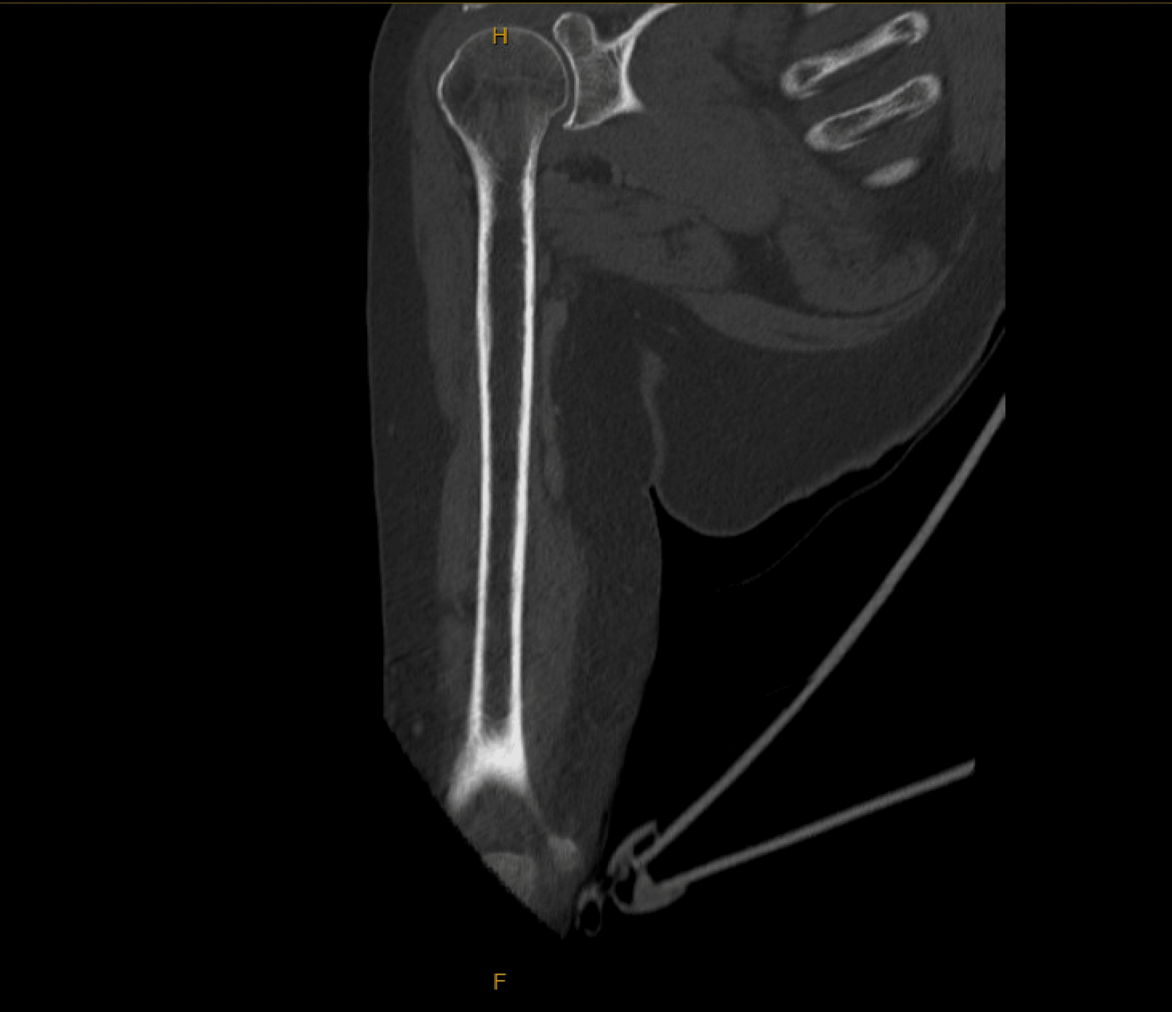
Coronal CT Bone Window Image of the Right Humerus With IV Contrast Coronal CT image of the right humerus in bone window (with IV contrast), demonstrating preserved alignment of the glenohumeral joint with mild-to-moderate joint space narrowing and subchondral changes consistent with osteoarthritis. The humeral head and glenoid are intact with no evidence of fracture, dislocation, or osseous lesion. The acromioclavicular joint is partially visualized superiorly and appears moderately degenerated. The visualized bony structures of the humeral shaft are intact, with no cortical disruption.

**Figure 3:**
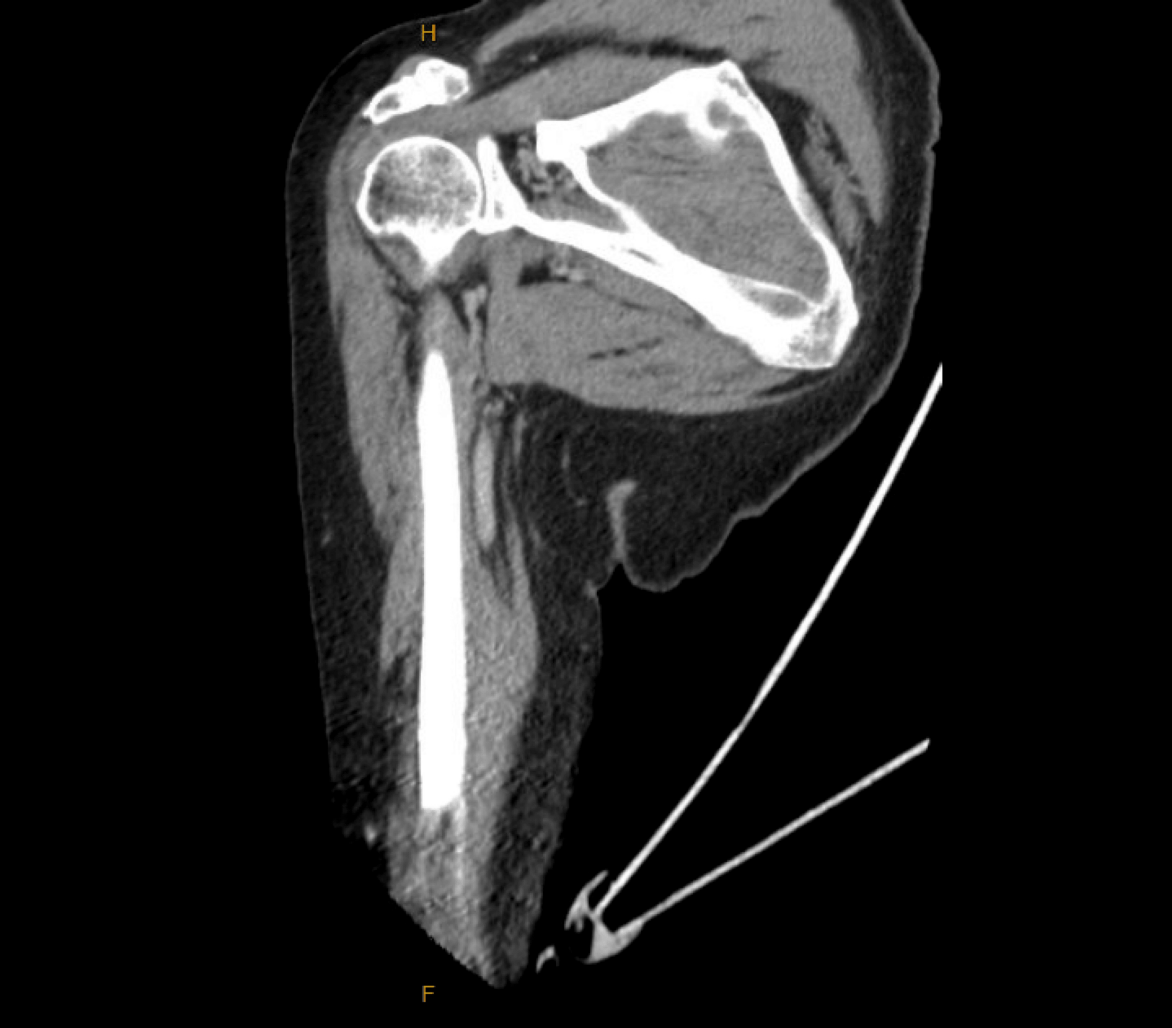
Coronal CT Soft Tissue Window Image of the Right Humerus With IV Contrast Coronal CT image of the right humerus in soft tissue window (with IV contrast), demonstrating preserved soft tissue planes and musculature without evidence of acute pathology. The deltoid and rotator cuff muscles appear intact with normal bulk and symmetry. No soft tissue mass, organized fluid collection, or joint effusion is visualized. The axillary region shows no lymphadenopathy, and the neurovascular bundle appears unremarkable. The acromioclavicular and glenohumeral joints are partially visualized and demonstrate chronic degenerative changes without acute abnormalities.

**Figure 4:**
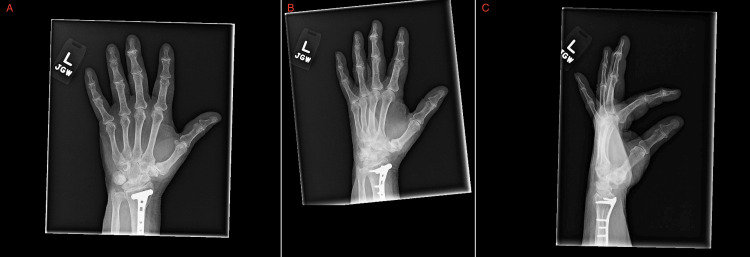
Multiview Radiographs of the Left Hand Demonstrating Chronic Degenerative Changes Anteroposterior (A), oblique (B), and lateral (C) radiographic views of the left hand demonstrate findings consistent with chronic degenerative joint disease. There is a partially visualized distal radial fixation plate with screws, consistent with prior surgical intervention. Mild joint space narrowing is seen at the radiocarpal joint. Notably, there is moderately severe joint space narrowing with osteoarthritic changes at the first carpometacarpal (CMC) joint, consistent with thumb base osteoarthritis. Additional degenerative changes are observed at multiple distal interphalangeal (DIP) joints, most pronounced in the third digit. No acute fracture, dislocation, or soft tissue abnormality is identified.

Imaging studies were obtained alongside lab testing. CT of the right humerus with intravenous contrast (Figures 1-3) showed no acute osseous or soft tissue abnormalities, mild-to-moderate glenohumeral joint OA, and moderate-to-severe acromioclavicular joint OA. CT of the right forearm with contrast showed no acute findings. A left hand X-ray (Figure 4) revealed a partially visualized distal radius fixation plate and screws, mild radiocarpal joint space narrowing, moderately severe degenerative changes at the first carpometacarpal joint, and degenerative changes in the distal interphalangeal (DIP) joints, most pronounced at the third DIP joint. No acute fractures or dislocations were seen.

In the days following her initial clinic visit, the patient developed acute swelling and pain in the right wrist and hand, particularly localized to the thenar region. She was evaluated at an urgent care center, where right hand radiographs were unremarkable. She was advised to rest, elevate the hand, and wear a wrist brace; no new medications were prescribed. Several days later, at follow-up, she reported modest improvement in right wrist symptoms. She denied systemic symptoms including fever, chest pain, fatigue, dyspnea, or gastrointestinal or urinary complaints.

On repeat examination, right wrist swelling was evident, with mild erythema over the volar surface but no appreciable warmth. The left wrist remained tender without swelling or erythema. The remainder of the physical examination was unremarkable.

Given the migratory pattern of joint involvement and weakly positive anti-CCP antibodies with very mildly elevated CRP in an otherwise negative work-up, a working diagnosis of early RA with an atypical migratory polyarthritis presentation was made. This diagnosis was supported by the patient's score when using American College of Rheumatology/European League Against Rheumatism (ACR/EULAR) 2010 Rheumatoid Arthritis Classification Criteria. Her involvement of four small joints with additional large joints, low positive anti-CCP antibodies, and slightly abnormal CRP gave her a score of 6, which the criteria interpret as "definitely RA" [7]. Crystalline, infectious, and vasculitic etiologies were considered but deemed less likely based on clinical, serologic, and imaging findings. The patient was started on a five-day course of oral prednisone 40 mg daily, resulting in partial symptom improvement. Initiation of methotrexate and folic acid was discussed; however, the patient elected to defer disease-modifying therapy pending reassessment of her response to corticosteroids. She was referred to rheumatology for further evaluation and long-term management.

## Discussion

This case illustrates the diagnostic complexity of rheumatoid arthritis (RA), particularly when it presents with an atypical migratory pattern. The patient’s shifting joint involvement initially suggested a broad differential diagnosis, including inflammatory and autoimmune conditions such as reactive arthritis, systemic lupus erythematosus (SLE), and inflammatory bowel disease (IBD)-associated arthritis and other conditions such as fibromyalgia (Table 2). Given the overlap in clinical features among these disorders, establishing a definitive diagnosis required careful correlation of symptoms, laboratory findings, and imaging results.

**Table 2:** Key Factors of Differential Diagnoses Considered and Reasoning for Their Ruling Out Typical features for leading differential diagnoses and reasons that each was ruled out are discussed. CRP: C-reactive protein, ESR: erythrocyte sedimentation rate, SLE: systemic lupus erythematosus, ANA: antinuclear antibodies, IBD: inflammatory bowel disease, GU: genitourinary, dsDNA: double-stranded DNA.

Disease	Typical Features	Reasons to Rule Out
Reactive arthritis	•Asymmetric oligoarthritis, often in the lower extremities •Preceded by GI or GU infection •May include systemic manifestations such as conjunctivitis or uveitis, urethritis or cervicitis, enthesitis, dactylitis, and mucocutaneous lesions •Positive HLA-B27 in 30%-50% [8]	•No preceding infection •Symmetric polyarthritis and migratory pattern, which is less typical •Absence of extra-articular features •Mild elevation of CRP and normal ESR
SLE	•Multi-system involvement (skin, kidneys, CNS, blood cells) •Non-erosive, often symmetric small joint arthritis •Associated with autoantibodies (ANA, anti-dsDNA) •Predominantly affects young women [9]	•Absence of systemic symptoms (e.g., rash, oral ulcers, serositis, cytopenias) •Negative ANA and other lupus-specific antibodies •Erosive and deforming joint abnormalities not commonly seen in SLE •Lack of renal, neurological, or hematological abnormalities
IBD-associated arthritis	•Associated with Crohn's disease or ulcerative colitis •Usually coexists with sacroiliitis or ankylosing spondylitis •Morning stiffness •Axial or peripheral involvement [10]	•No known history or symptoms of IBD (e.g., chronic diarrhea, abdominal pain, rectal bleeding) •No sacroiliac tenderness or back stiffness
Fibromyalgia	•Chronic widespread musculoskeletal pain •Fatigue, non-restorative sleep, cognitive complaints •Tender points on exam •Often associated with anxiety, depression, IBS •Normal inflammatory markers •Often seen in middle-aged women [11]	•Objective signs of inflammation (e.g., joint swelling, warmth) •Mildly elevated CRP •Migratory pattern more consistent with inflammatory arthritis •Positive anti-CCP functional limitations due to joint pathology, not just pain sensitivity

A key challenge in diagnosing RA is the variability of serologic markers [3]. While rheumatoid factor (RF) has traditionally been used in diagnosis, it is not specific to RA and may be positive in other disease conditions, such as Sjögren's syndrome, ANCA-associated vasculitis, and COVID-19 [12-14]. In terms of clinical utility, the test has a specificity of approximately 85%, a sensitivity of 69%, a positive predictive value of 4.86, and a negative predictive value of 0.38 [15]. In contrast, anti-cyclic citrullinated peptide (anti-CCP) antibodies are more strongly associated with RA and are considered a more specific and definitive marker of the disease [15,16]. Anti-CCP antibodies have a specificity of 95%, sensitivity of 67%, positive predictive value of 12.46, and a negative predictive value of 0.36 [15]. In this case, the patient’s weakly positive anti-CCP antibodies, number of small and large joint involvement, and mildly elevated CRP strongly supported a diagnosis of RA, based on ACR/EULAR 2010 Rheumatoid Arthritis Classification Criteria, despite her negative RF status [7].

Initial treatment with corticosteroids led to partial symptom improvement, further supporting an inflammatory, rather than purely degenerative, etiology. While the patient deferred initiation of methotrexate, early use of disease-modifying antirheumatic drugs (DMARDs) remains the standard of care to slow disease progression and prevent joint destruction [1,3]. Current clinical recommendations for second-line agents are biologic agents such as Janus kinase (JAK) inhibitors, sulfasalazine, and hydroxychloroquine [17]. This case underscores the necessity of a comprehensive approach in evaluating patients with complex joint symptoms and highlights the critical role of anti-CCP antibodies in distinguishing RA from other rheumatologic diseases.

Another complicating factor in this case was the patient’s advanced age, obesity, and pre-existing osteoarthritis (OA) [18]. Given the high prevalence of OA in elderly individuals, there is a risk of attributing all joint-related symptoms to degenerative changes, thereby overlooking an underlying inflammatory pathology [19]. However, OA alone does not typically cause the migratory and inflammatory joint symptoms observed in this patient [1-3]. Instead, her OA may have masked the presence of inflammatory arthritis, delaying consideration of an alternative diagnosis. This highlights the importance of maintaining a high index of suspicion and not dismissing evolving symptoms based solely on pre-existing degenerative joint disease [20].

Comorbid OA is present in approximately 44% of patients with RA and has been associated with poorer rheumatologic care and worse clinical outcomes [19]. The overlapping clinical features of these conditions can lead to missed diagnoses and suboptimal management [19]. Given the importance of integrating clinical evaluation with serologic testing in the diagnostic workup of RA and the high rate of comorbid OA, the diagnostic approach should account for the possible coexistence of multiple autoimmune or degenerative conditions [19]. Doing so can help prevent diagnostic delays and improve patient outcomes. Because RA does not always follow a classic clinical presentation and is often comorbid with other conditions, clinicians should avoid excluding the diagnosis based on any single criterion.

## Conclusions

This case underscores the importance of maintaining a broad differential and a high index of suspicion for rheumatoid arthritis (RA), especially when symptoms deviate from the classic presentation. Migratory joint pain, although less common, can be an early manifestation of RA and is often misattributed to mechanical or degenerative conditions, particularly in older adults with known osteoarthritis (OA).

Positive anti-cyclic citrullinated peptide (anti-CCP) antibodies played a pivotal role in establishing the diagnosis in this seronegative patient, highlighting their diagnostic value in atypical presentations. Prompt identification and initiation of appropriate therapy, including early use of disease-modifying antirheumatic drugs (DMARDs), are essential to preserving joint function and preventing systemic complications. Clinicians should remain aware of the broad spectrum of RA presentations and include it in the differential diagnosis, even in patients with coexisting rheumatologic or degenerative conditions, to ensure timely diagnosis and management.
